# A High Performance Cloud-Based Protein-Ligand Docking Prediction Algorithm

**DOI:** 10.1155/2013/909717

**Published:** 2013-05-14

**Authors:** Jui-Le Chen, Chun-Wei Tsai, Ming-Chao Chiang, Chu-Sing Yang

**Affiliations:** ^1^Department of Electrical Engineer, National Cheng Kung University, Institute of Computer and Communication Engineering, Tainan 70101, Taiwan; ^2^Department of Digital Multimedia Design, Tajen University, Pingtung 90741, Taiwan; ^3^Department of Applied Informatics and Multimedia, Chia Nan University of Pharmacy & Science, Tainan 71710, Taiwan; ^4^Department of Computer Science and Engineering, National Sun Yat-sen University, Kaohsiung 80424, Taiwan

## Abstract

The potential of predicting druggability for a particular disease by integrating biological and computer science technologies has witnessed success in recent years. Although the computer science technologies can be used to reduce the costs of the pharmaceutical research, the computation time of the structure-based protein-ligand docking prediction is still unsatisfied until now. Hence, in this paper, a novel docking prediction algorithm, named fast cloud-based protein-ligand docking prediction algorithm (FCPLDPA), is presented to accelerate the docking prediction algorithm. The proposed algorithm works by leveraging two high-performance operators: (1) the *novel* migration (information exchange) operator is designed specially for cloud-based environments to reduce the computation time; (2) the *efficient* operator is aimed at filtering out the worst search directions. Our simulation results illustrate that the proposed method outperforms the other docking algorithms compared in this paper in terms of both the computation time and the quality of the end result.

## 1. Introduction

 The ultimate goal of most people is looking for every possible solution that provides a more comfortable life; therefore, most researchers have done their best to advance the interest of human from different positions, domains, concerns, and backgrounds. One important work for the lighthearted life is finding a new drug for particular disease. Needless to say, drug design can always help human health because it can be used in preventing and curing diseases. The structure-based drug design [1] usually can be used to predict the interactions between small drug molecules and protein receptor complexes, and now, it is one of the well-known computer-aided drug design methods. With advance of computer technologies, the prediction method based on theoretical computing method and molecular modeling to establish the three-dimensional structure for designing a new drug molecule can be used to speed up finding the good possible candidate solutions. As observed by Volkamer et al. [2], even though we invest more than one thousand billion US dollars for drug development, the prediction accuracy and the development time are still unsatisfied. In other words, the prediction accuracy of the docking prediction is no more than 70% while the drug discovery process still takes a tremendous amount of computation time just to find the possible drugs.

 To measure the simulation results, the Van der Waals (VDW), atomic radius, charge, torsional angles, intermolecular hydrogen bonds, and hydrophobicity of the contact force are usually used to bind the energy between receptor and ligand. The empirical energy function [3], such as the score function, is usually used to evaluate the results of ligand molecular docking conformation which is suitable or not for binding area of receptor. Each candidate solution of the protein-ligand docking prediction (PLDP) problem contains the three-dimensional coordinates of the ligand center point, the four orientation parameters, and some additional special atoms, such as coal, nitrogen, and hydrogen whose free torsion degrees are used as the parameters. The set of candidate solutions *X* can be expressed as the total energy of the protein-ligand interaction and the sum of the internal energy for both ligand and protein which is given as follows:

(1) $$\begin{array}{c} \min\;E_{\text{total}}\left(X\right)=E_{v}+E_{h}+E_{e}+E_{i}+E_{d}, \end{array}$$

where *E*_*v*_, *E*_*h*_, and *E*_*e*_ denote, respectively, the interaction forces of intermolecular, namely, Van der Waals forces, hydrogen bond, and electronic potential energy; *E*_*i*_ is the internal attraction of ligand and protein molecules; *E*_*d*_ is the desolvation of binding area meaning the performance for hydrophobic.

Because the search space of possible conformations is extremely large, how to reduce the computation time has become a very important research issue, especially that all these problems are usually either NP-hard or NP-complete problem [4]. Hence, a high-performance search method is required to speed up the overall performance of the search process. This explains why many search methods for reducing the computation time have been presented to solve the docking problem [5]. The heuristic algorithms, such as simulated annealing (SA) [6] and genetic algorithm (GA) [3, 7], provide a fast method to search for approximate solutions which are faster than the brute force search algorithms and traditional search algorithms. As such, it is one of the efficient ways for solving the docking problem [8].

To enhance the performance of heuristic algorithms for the docking problem, this paper presents a novel protein-ligand docking prediction algorithm to speed up the process of drug design and development on a *cloud computing* environment, by using a novel migration method while at the same time attempting to improve the accuracy rate (success rate) of prediction by using an efficient operator to filter out the worst search direction.

The rest of the paper is organized as follows. In Section 2, a brief introduction to the parallel computing for the protein-ligand docking prediction problem is given. After that, the concept and design of the proposed algorithm are detailed in Section 3.  Section 4 begins with a brief description of the materials presented in this paper and then compares the simulation results of the proposed algorithm with those of other protein-ligand docking prediction algorithms. The conclusion is drawn in Section 5.

## 2. Related Work

In addition to using metaheuristics to improve the performance of the docking prediction algorithm as we mentioned in Section 1, another way is to enhance the computation power of hardware, such as parallel computing. However, since the communication and synchronization costs of the search algorithm for PLDP on a cloud computing environment are much higher than those on a grid or cluster computing environments, to enhance the performance of the protein-ligand docking prediction process, we have to take into consideration these factors in the design and development of protein-ligand docking prediction algorithm (PLDPA).

Among others, three major parallel computation models are usually used in the evolutionary computation and other metaheuristics [9–11] for enhancing the search performance, to not only cut down the computation time but also improve the quality of the end result. These parallel computation models are master-slave model [9], fine-grained model (cellular model) [9], and coarse-grained model (island model) [11]. For instance, the master-slave model for genetic algorithm will divide the population into several subpopulations and then assign them to different processors to accelerate the computation speed. For the fine-grained model, it also divides the population into several subpopulations each of which are assigned to different processors or machines. But each subpopulation can only exchange information with other subpopulations to which they are directly connected. The coarse-grained model (also called the island model) uses the concept of island and migration to exchange the information between subpopulations. Unlike traditional computing approaches, the parallel computation models take into account both the architecture of the search algorithm and the computation resources together. The major concerns now become how the chromosomes communicate and exchange information between the subpopulations to affect their search performance [12–15].

For the protein-ligand docking prediction (PLDP), Wang et al. [16] use the master-slave model for Lamarckian genetic algorithm (LGA) (one kind of hybrid genetic algorithm for which the genetic algorithm (GA) plays the role of global search while the local search algorithm plays the role of fine-tuning the search results found by GA) to speed up the computation time of the docking prediction process. Various successful works have been presented in recent years. For instance, Kannan and Ganji [17] used GPU to speed up the search process of PLDP. Sampling methods [18] have been employed to provide not only better initial seeds but also the possibility of finding better results. Some researchers [19, 20] attempted to redesign or modify the scoring function for PLDP because the scoring function takes a large percentage of the computation time [17]. These researches focus on either reducing the computation time, increasing the accuracy of prediction, or both.

**Figure 1:**
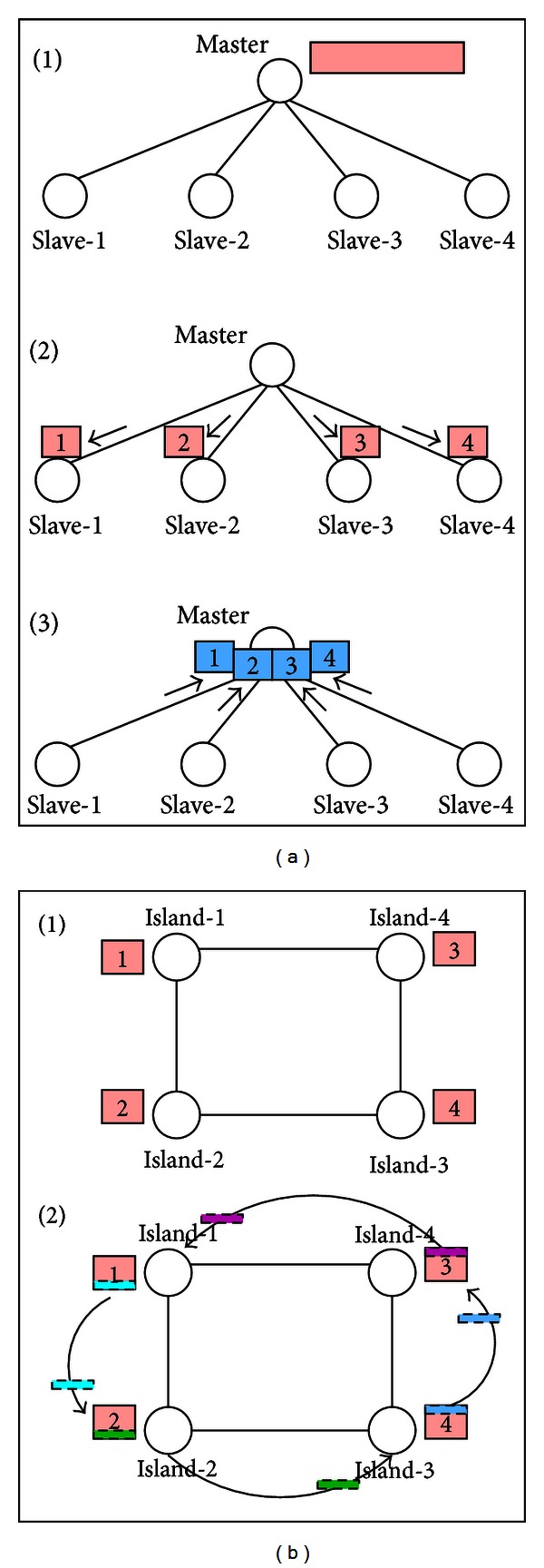
A simple example for illustrating the parallel computation models. (a) Master-slave model; (b) island model.

As a promising research area in recent years (after the grid [21] and cluster [22, 23] system), cloud computing provides a better way to enhance the performance of PLDP, such as a tremendous amount of the compute and storage resources, which leverages the strengths of grid computing and cluster computing. How to apply the PLDP algorithm to this new infrastructure have nowadays become a critical research issue.  Figure 1 gives the details of master-slave model and island model. For these distributed computing models, the main concern is how to divide the computations of a search algorithm and then dispatch them to the computer nodes to improve the search performance.

## 3. The Proposed Method

### 3.1. Concept

Since most heuristic algorithms do not guarantee that they can find the optimal solution, one of the most important problems for the heuristic algorithms to deal with is to balance the computation cost and the quality of the solution. For the cluster computing environment, most computer resources (nodes) are centralized in the same place; therefore, the communication and synchronization costs are not as high. For grid and cloud computing environments, they are, however, an important issue because most computing nodes are not centralized in the same place. Also, from the perspective of algorithm design, because the total computation time of each slave (or island) is different, no matter which of the parallel computation models is used, it is almost unavoidable to waste time waiting for the other slaves to finish their tasks.

The proposed algorithm integrates two efficient operators. The first one is a novel migration operator to mitigate the costs incurred on a cloud-based environment whereas the second operator is the pattern reduction operator [29, 30] to filter out the worst search directions. Just like other researches on protein-ligand docking prediction and [29], the main focus of this research is not only on the development of a faster search process but also on getting better solutions for the binding locations of the protein-ligand docking prediction.

### 3.2. The FCPLDPA

As shown in Algorithm 1, the proposed algorithm (FCPLDPA) is applied to the Lamarckian genetic algorithm (LGA) to solve the docking problem for the rigid protein and flexible drug molecules. The FCPLDPA will first construct the initial solution *S* and then divide it into *m* subpopulations (the number of *m* is predefined by the user, which usually matches the number of virtual machines (computing nodes) that can be used for solving the docking prediction problem) *S* = {*S*_1_, *S*_2_,…, *S*_*m*_}. Next, each subpopulation will be dispatched to a virtual machine (island). Like the island model, each subpopulation will now undergo the evolution process independently from each other except that some of the chromosomes are *immigrations* or *emigrations* of the island. Unlike the classical island model, the migrations of the proposed algorithm are not restricted to be at the same iteration number (era) because such a restriction may delay the migration process, by waiting for the other islands to converge.  Algorithm 2 gives the details of the evolution process for the islands and the migration procedure, which include the evolution process of the simple genetic algorithm—selection, crossover, and mutation operations. However, in addition to applying the pattern reduction operator [29] (as shown in line 5 of Algorithm 2) to the proposed algorithm, the timing for migrating the chromosomes to the other islands (virtual machines) is the main concern of this paper, as shown in lines 7 and 10 of Algorithm 2. The migration mechanism is as given below:

(2) ψ(Ti,ℱi,ℳi) ={1if  𝒯i=𝒯H,  ℱi=false2if  𝒯i=𝒯H,  ℱi=false,  ℳi=true,0otherwise,

where *M*_*T*_ denotes the timing (i.e., migration interval) to synchronize all the islands (virtual machines), *T*_*i*_ is the number of iterations that has been performed on the evolution process of the *i*th virtual machine (VM), *𝒯*_*H*_ is the threshold to determine the timing to migrate the chromosomes to the other islands, *ℱ*_*i*_ is the flag to show whether any migration process has been done or not since the previous migration process was performed, and *ℳ*_*i*_ is the policy of master to determine the timing to exchange information between islands. More precisely, because the migration process of the islands occurs for particular islands (as shown in Figure 1, this means that some of the islands will complete their tasks at about the same time). The synchronization process is needed to be performed after some of the migration processes (or once every *M*_*T*_ iterations) so that the proposed algorithm is able to transmit the information from one island to the others. Note that *M*_*T*_ is defaulted to 25, and *T*_*i*_ is defaulted to 5. 

In the *case of emigration*, that is, the first case in (2), it illustrates that the migration process will be performed when the number of iterations at the *i*th island is equal to *T*_*H*_ and *F*_*i*_ is false. In this case, the proposed algorithm will select a chromosome (elite solution) to migrate to the other islands, just like the island model of GA. However, in the *case of immigration*, that is, the second case in (2), any chromosome that wants to enter the *i*th island must also satisfy the condition *T*_*i*_ = *T*_*H*_ (i.e., after *T*_*H*_ iterations). The master needs to choose the islands the evolution processes of which are completed at about the same time to exchange the information. It will become time oriented; thus, most of the migration processes need not to wait for the evolution processes of the other islands to finish.

**Figure 2:**
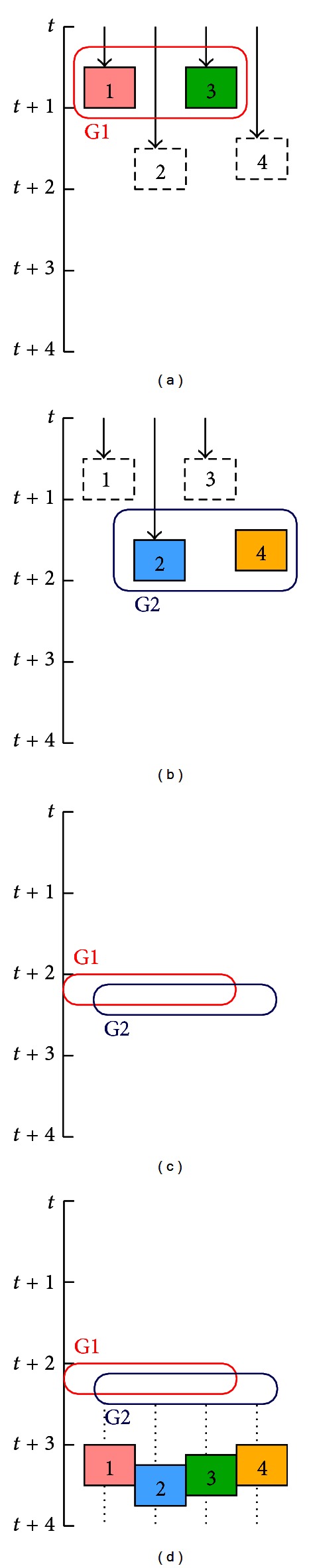
A simple example illustrating how the proposed algorithm works. (a) island-1 and island-2 complete their work at time *t* + 1; (b) island-2 and island-4 complete their work at about time *t* + 2; (c) after the exchange of information between all the islands, FCPLDPA will synchronize the information; (d) and then FCPLDPA will continue to let all the islands exchange information with approximate island.

A simple example is given in Figure 2 to explain the main idea of the migration strategy of the proposed algorithm.  Figure 2(a) shows that not all the islands will complete their work at the same time if the time for migration is set up to, say, once every five iterations. The problem of all the islands not being able to finish the same work at the same time is owing to two important factors. One is due to the communication cost (including the synchronization and other transmission costs) while the other is due to the randomness of the convergence speed of most metaheuristics. The proposed algorithm attempts to deal with this problem, by letting islands exchange the information once they completed their work at about the same time. A very simple method is to let island-*b* exchange information with island-*a* if the completion time of island-*b* is closest to that of island-*a* and they have not exchanged information to the other islands in this information exchange round (i.e., over the past five iterations). Similar to Figure 2(a), Figure 2(b) also lets island-2 and island-4 exchange information (chromosome migration) at time *t* + 2 because their completion times are closest to each other.

Figure 2(c) shows that when all the islands have exchanged their information after *M*_*T*_ iterations that is, migrate the chromosomes to other islands, the proposed algorithm will then perform a synchronization procedure. That is, it will randomly pick the chromosomes from these groups (island-1 and island-3 as the first group while island-2 and island-4 as the second group) and migrate them to islands of the other group so that the information can be circulated to all the islands. After that, as shown in Figure 2(d), FCPLDPA will continuously let all the islands evolve their subpopulations again and migrate their chromosomes to the other islands later. In summary, if the communication costs are high and the convergence speeds of all the islands are not the same, the proposed algorithm can be used to avoid wasting time to wait for the other islands to complete their work. The detailed analysis will be given in later sections.

## 4. Simulation Results

In this paper, the performance of the proposed algorithm is evaluated by using it to solve the protein-ligand docking problem. All the empirical analyses are conducted on 16 VMs, each of which consists of one CPU (2.5 GHz), 4 GB of memory, a 100 MBps NIC card, and GNU C++ v4.12, and runs CentOS_64 v6.2. Also, the test platform for docking is AutoDock 4.2 [31].

Several state-of-the-art algorithms, namely, differential evolution [32], particle swarm optimization [33], Lamarckian genetic algorithm with island model (parallel GA; PGA) [34, 35], FCPLDPA without PR, and FCPLDPA with PR, are applied to the AutoDock environment to search for the possible protein-ligand binding sites. The energy function which calculates the energy value between the protein and ligand molecule is used by these search algorithms to determine which conformation is the candidate with the most appropriate binding points. The details of composition formula for free energy expressions could be referred to the study of [3]. For all these algorithms, the population size *N* is fixed at 256, the subpopulation size for the parallel model is fixed at *N*/*I*_*n*_ where *I*_*n*_ denotes the number of islands, the crossover rate is set equal to 0.9, the mutation rate is set equal to 0.08, and the number of generations *ℓ* is set equal to 10,000.

### 4.1. Materials

**Figure 3:**
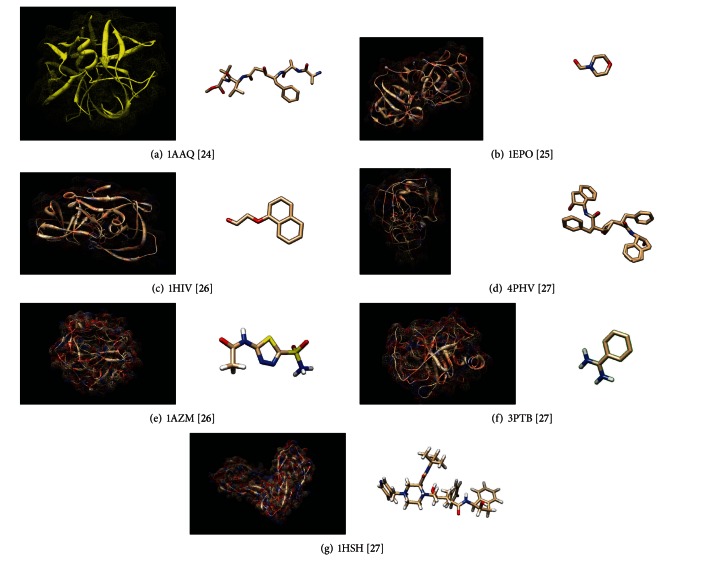
Structure diagrams [28] for both protein and ligand molecules.

The molecular docking problem can be regarded as the key matching problem where the lock and key are the receptor and ligand, respectively, and the goal of all the simulations is to provide an efficient way to find the approximate positions of the key and lock from a large search space or the candidate set of drugs. Although until now, the accuracy of most candidate sets of approximate positions (solutions) found by a search algorithm is not as precise as it is supposed to be; it does provide an efficient way to find out a good drug molecule which is not validated yet by a laboratory. In this paper, an effective tool for drug design based on structure-based protein molecule, AutoDock [6], is used to evaluate the proposed algorithm and the other state-of-the-art docking prediction algorithms, such as GA and parallel GA (PGA). In addition, four different kinds of data sets, as shown in Figure 3, are used to evaluate the performance of the proposed algorithm and the state-of-the-art docking prediction algorithms compared in this paper. The data sets are taken from the RCSB Protein Data Bank database (http://www.pdb.org/).

### 4.2. Results

**Figure 4:**
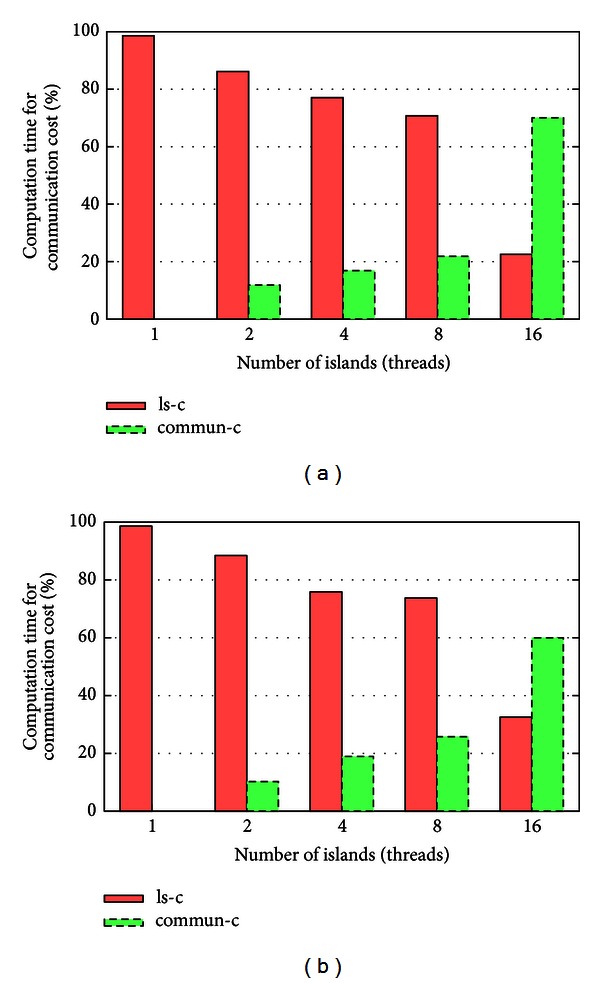
Percentage of the time due to communication (commun-c) and local search (ls-c) while the number of islands represents LGA. (a) 1AAQ; (b) 1EPO.

Our observation shows that most computation costs of LGA come from the local search process. But this characteristic will be changed for the parallel GA on a cloud computing environment. As shown in Figure 4, two interesting phenomena can be easily observed. First, the communication costs will increase as the number of islands (virtual machines) increases. These results show that the communication costs and the convergence speed of different islands all may affect the performance of the system. Second, the local search process may change the percentage of the computation time for all the operators of protein-ligand docking prediction. Figures 4(a) and 4(b) show that the local search of PGA takes much more computation time than the function evaluation, especially when we invest much more resources to the local search process for the four different datasets which differs from the observation described in [17] because Kannan and Ganji believe that the function evaluation takes most of the computation time of the whole convergence process. However, our observation is that the function evaluation will not affect the computation time of the whole search process. These results help us emphasize that the local search, the communication cost, and the different convergence speed of all the islands are the other important factors to be taken into account for the computation time of protein-ligand docking prediction, especially when we are using the cloud computing environment to solve the protein-ligand docking prediction problem.

As shown in Table 1, seven different datasets are used to evaluate the performance of the proposed algorithm and the other docking prediction algorithms. For each algorithm, the table gives the percentage of time taken by the initialization, selection, crossover, mutation, function evaluation, local search, and send and receive operators. The results show that the proposed algorithm outperforms the PGA in most cases, either without PR or with PR in terms of the success rate and the average time.

Comparison of the proposed algorithm with PGA shows that if the send and receive costs can be decreased, the overall computation time can also be decreased. According to our observation, FCPLDPA without PR can provide a better success rate than PGA and DE because the proposed algorithm postpones the transmission of information from the island to all the other islands; therefore, the search diversity between islands can be maintained. Another strategy for the proposed algorithm is to combine it with PR, called FCPLDPA with PR (FCPLDPA + PR). The simulation results in Table 1 also show that FCPLDPA + PR can provide better results than DE, PSO, PGA, and FCPLDPA alone in terms of the success rate with a little more investment of the computation time.

## 5. Conclusion

 In this paper, a novel docking prediction algorithm named fast cloud-based protein-ligand docking prediction algorithm (FCPLDPA) is presented to enhance the performance of metaheuristics (i.e., GA-based algorithm) for pharmaceutical research. The simulation results show that the proposed algorithm can not only significantly reduce the computation cost of GA in solving the protein-ligand docking prediction problem by using cloud computing technologies but also improve the quality of the end result by using the pattern reduction method. They also show the possibility of using cloud computing technologies and the dilemmas we need to face when applying the drug prediction approaches to the cloud computing environment. More precisely, many approaches can be used to reduce the computation costs of metaheuristics, such as investing more computing resources to finish the job faster or using better search strategy (i.e., sampling or dimension reduction methods). However, according to our observation, the improvement was not proportional to the investment because the communication costs and the different convergence speeds of virtual machines (islands) all affect the performance of the docking system on cloud. The main purpose of this research is to eliminate the *waiting between different virtual machines* of cloud-based docking prediction algorithm. The simulation results are consistent with our assumptions and observations that the communication costs and the different convergence speeds between islands may strongly impact the performance of the purposed algorithm. Two efficient operators are employed in this paper: (1) the novel migration operator is aimed to avoid wasting of the computation power and waiting for the other virtual machines on a cloud computing environment, and (2) the pattern reduction operator is aimed to enhance the search performance. The main contributions of this research can be summarized as follows: (1) we discovered that the communication costs and the different convergence speeds between virtual machines (islands) will eventually affect the performance of the search algorithm on cloud; and (2) we presented a high-performance cloud-based protein-ligand docking prediction algorithm to deal with this problem to guide the search algorithm to find the approximate candidate solution quickly. In the future, we will focus on finding a more efficient prediction method to improve the accuracy of the solution of FCPLDPA while reducing the computation time of the whole process.

## Figures and Tables

**Algorithm 1:**
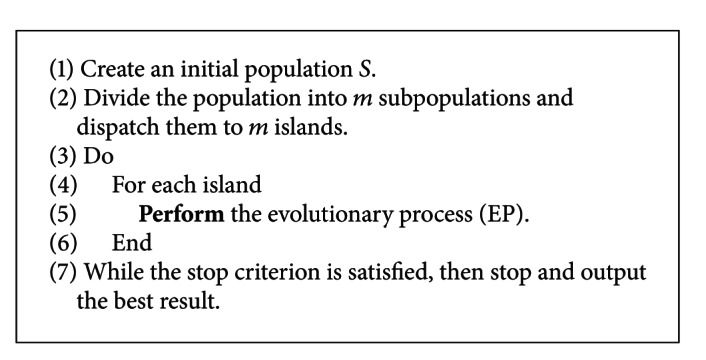
Outline of FCPLDPA for the protein-ligand docking prediction problem.

**Algorithm 2:**
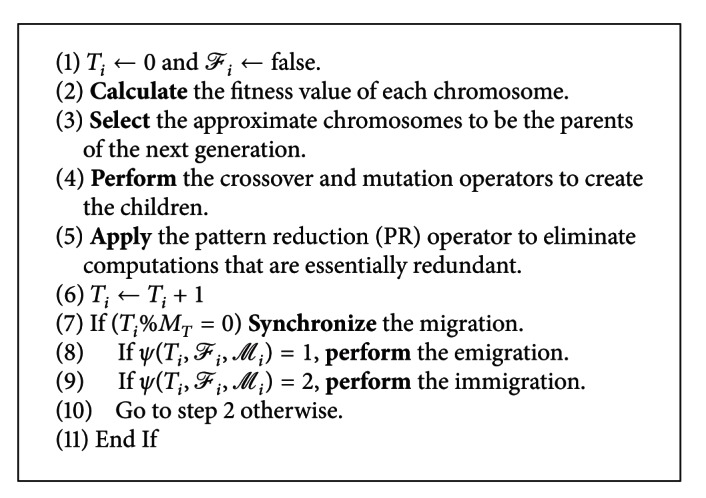
Outline of the evolutionary process (EP).

**Table 1:** Comparison of the proposed algorithm with the other docking prediction algorithms.

Islands	1	2	4	8	16	32
Chromosomes	256	128	64	32	16	8
DE (island model)						

Initialization	0.11%	0.13%	0.34%	0.53%	1.34%	2.63%
Mutation	2.39%	2.44%	2.36%	2.37%	2.76%	2.72%
Evaluation	0.61%	0.71%	0.68%	0.66%	0.77%	0.64%
Local search	96.88%	93.92%	90.13%	82.86%	70.59%	48.69%
Send and receive	0.00%	3.51%	7.17%	14.24%	23.31%	43.96%

Success rate	22.86%	27.14%	30.71%	33.57%	40.71%	39.29%
Average time	1,726.52	995.23	506.34	251.93	154.33	104.07

PSO (island model)						

Initialization	0.07%	0.14%	0.26%	0.78%	1.56%	3.12%
Evaluation	2.37%	2.75%	2.74%	2.11%	3.28%	2.94%
Local search	97.55%	95.44%	94.61%	90.96%	82.4%	52.69%
Send and receive	0.00%	1.68%	2.39%	6.15%	13.02%	26.14%

Success rate	17.86%	21.43%	25.00%	31.43%	35.71%	32.14%
Average time	284.41	176.39	87.39	42.94	28.15	18.78

PGA (island model)						

Initialization	0.20%	0.29%	0.68%	0.94%	0.90%	0.90%
Selection	0.20%	0.34%	0.68%	0.94%	0.90%	0.90%
Crossover	0.01%	0.01%	0.01%	0.01%	0.01%	0.01%
Mutation	0.01%	0.01%	0.01%	3.86%	0.10%	0.01%
Evaluation	0.98%	1.69%	3.41%	4.72%	4.44%	4.50%
Local search	98.63%	88.95%	76.72%	62.57%	26.22%	22.20%
Send and receive	0.00%	8.98%	18.29%	28.50%	67.28%	71.35%

Success rate	15.00%	19.29%	21.43%	28.54%	34.29%	27.14%
Average time	572.74	330.71	168.07	84.33	50.14	33.59

FCPLDPA without PR						

Initialization	—	0.34%	0.68%	0.94%	0.90%	0.90%
Selection	—	0.34%	0.68%	0.94%	0.90%	0.90%
Crossover	—	0.01%	0.01%	0.01%	0.01%	0.01%
Mutation	—	0.01%	0.01%	1.29%	0.01%	0.01%
Evaluation	—	1.78%	3.74%	5.26%	5.19%	5.89%
Local search	—	93.35%	84.13%	69.61%	30.69%	26.67%
Send and receive	—	4.43%	10.41%	19.42%	61.69%	65.61%

Success rate	—	25.00%	25.71%	30.71%	37.14%	32.14%
Average time	—	313.49	153.78	75.90	42.69	30.54

FCPLDPA with PR						

Initialization	—	0.34%	0.68%	0.86%	0.90%	0.90%
Selection	—	0.31%	0.68%	0.83%	0.90%	0.90%
Crossover	—	0.01%	0.01%	0.01%	0.01%	0.01%
Mutation	—	0.01%	0.01%	1.43%	0.01%	0.01%
Evaluation	—	1.78%	3.75%	5.02%	4.06%	5.96%
Local search	—	93.20%	83.59%	70.60%	32.66%	28.50%
Send and receive	—	4.48%	10.24%	20.88%	61.17%	64.09%

Success rate	—	27.86%	32.14%	40.00%	47.14%	45.71%
Average time	—	320.91	156.19	77.56	44.07	33.49
